# Hearing All About Donepezil: Its Role in the Field of Auditory Processing

**DOI:** 10.7759/cureus.93143

**Published:** 2025-09-24

**Authors:** Patra Sorod, Grace I Chen

**Affiliations:** 1 Geriatrics, University of California Los Angeles David Geffen School of Medicine, Los Angeles, USA

**Keywords:** alzheimer's, auditory, auditory hallucination, cognitive decline, dementia, donepezil, hearing loss

## Abstract

Donepezil is a central-acting acetylcholinesterase inhibitor aimed at increasing acetylcholine availability at the neuron synapses to enhance cholinergic transmission. It is often used to assist with cognitive function and is well-known as the first-line treatment for mild to severe dementia of several etiologies. However, its mechanism of action within “top-down” and “bottom-up” neurological pathways continues to be explored. This case reports on a 93-year-old woman in an ambulatory geriatrics clinic with moderate-to-severe dementia. Her significant history of hearing loss with auditory hallucination impressively responded to the initiation of donepezil. There may be potential benefits of donepezil within the peripheral pathways of neuro-cortical reorganization in the field of hearing loss and auditory processing.

## Introduction

Donepezil, approved for medical use in 1996, has been considered the first-line medication for treatment of mild to severe dementia of varying types, except for frontotemporal dementia. About 40-58% of users notice symptomatic improvement after starting donepezil to maintain cognition and functionality with the goal of delaying disease progression and institutionalization [1]. Adverse effects from donepezil and its varying effects on each patient are often the leading causes of its discontinuation. However, studies continue to demonstrate that compared to placebo, donepezil results in significant improvements in cognitive function, particularly in patients with early-stage Alzheimer’s disease [2,3].

Donepezil is a central-acting, rapid, reversible acetylcholinesterase inhibitor aimed at increasing acetylcholine (ACh) availability at the neuron synapses to enhance cholinergic transmission [4]. ACh is a neurotransmitter known to modulate different neurobiological processes in the brain. It promotes neuronal and synaptic plasticity to help activate attention, learning, memory, and cognitive reinforcement [5]. Neuronal synapses can travel two ways: “top-down” neurological responses stem from the brain to peripheral sensory predictions, while “bottom-up” responses collect peripheral sensory inputs to the brain for processing [6]. The understanding of the mechanism of action of donepezil along these pathways continues to be explored and can possibly provide benefits not only to treat dementia but also play a role in the auditory field.

## Case presentation

A 93-year-old woman presented to the geriatrics clinic with her spouse to establish care with primary concerns of cognitive decline. Her past medical conditions include right hip osteoarthritis, osteoporosis, recurrent urinary tract infections, and an extended history of prolonged hearing loss.

Her deafness started five years prior to presentation and had extended workup with audiology. She was diagnosed with bilateral sensorineural hearing loss and wore hearing aids for several years before becoming entirely deaf. Bone conduction implants were discussed but not pursued due to patient's osteoporosis. She communicates by reading lips, reading written words with her magnifying glass, or large font typed on the computer screen, to which she responds appropriately to facilitate conversation. Over time, patient's spouse noted that her memory slowly declined, with increasing difficulty with short-term memory, along with increased anxiety that included hallucinations and confusion every evening. Her spouse is able to reorient and redirect her during the day, but she wanders their home during the night stating she hears “buzzing in her head.” The “buzzing” is described as constant music often songs she has heard in her past. She enjoys humming along but other times, these tunes become noise disrupting her peace and sleep. It is unclear if the musical auditory hallucinations started before or after her cognitive decline.

**Figure 1:**
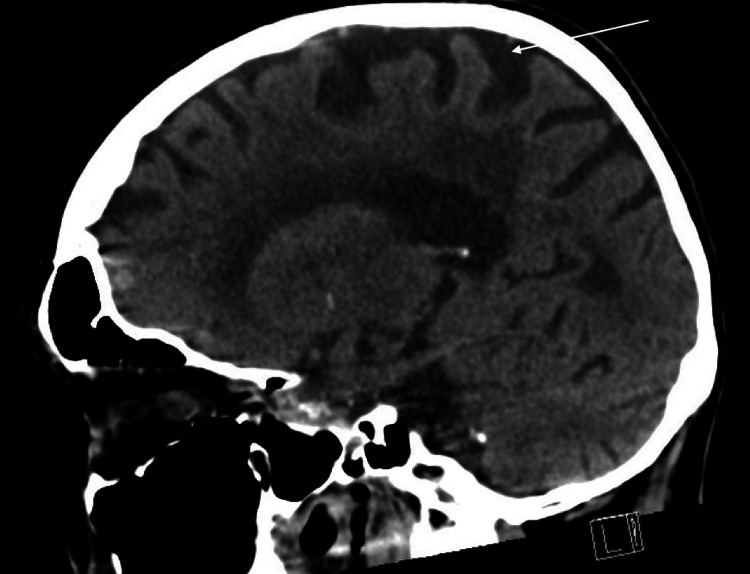
CT brain without contrast, sagittal view. Diffuse cerebral volume loss noted.

**Figure 2:**
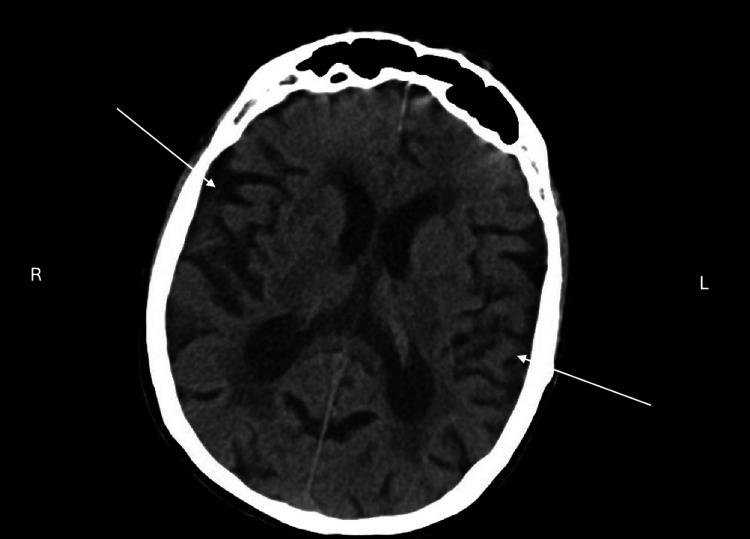
CT brain without contrast, transverse view. Diffuse parenchymal volume loss, more pronounced in the right greater than left bilateral anterior mesial temporal lobes.

**Figure 3:**
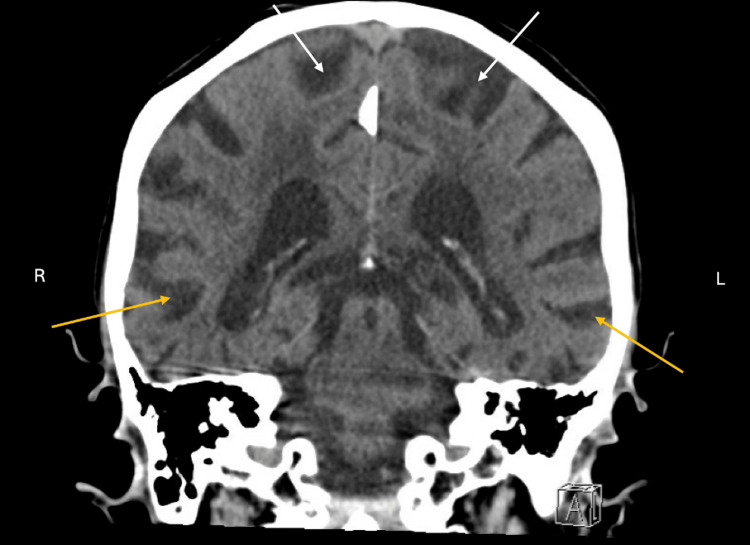
CT brain without contrast, coronal view. Diffuse cerebral volume loss was noted (white arrows) with more loss in the right temporal lobe compared to the left (yellow arrows).

Full assessment was completed for her cognitive impairment. Functional assessments demonstrated that she needed assistance with all her instrumental activities of daily living. She was able to complete some basic activities of daily living but required close monitoring for safety. She uses a front-wheel walker consistently both indoors and outdoors. Medications only included aspirin 81 mg daily and a multivitamin. Blood tests, including complete blood count, complete metabolic panel, thyroid function, and folate, were unremarkable (Table 1). Vitamin B12 was low and repleted. The urine test was positive for infection and was treated accordingly. Physical exam was unremarkable. There were no tremors, bradykinesia, or cogwheel rigidity. Sit-to-stand was intact with the assistance of her walker. She ambulated with and maneuvered the walker well with intact balance without shuffling or wobbly gait. Neurological exam was negative for focal neurological deficits. CT scan of the brain showed diffuse cerebral volume loss that appeared pronounced in the right anterior mesial temporal lobe compared to the left, as well as moderate small vessel ischemic changes (Figures 1-3). On the Montreal Cognitive Assessment (MoCA) [7] modified for hearing loss with the instructions provided in writing and lip reading, she scored 14/25, which converts to 16.5/30. Additional history obtained also revealed positive family history of Alzheimer’s disease.

**Table 1:** Lab results. GFR: glomerular filtration rate, TSH: thyroid-stimulating hormone.

Labs	Results	Reference range
White blood cell count	6.66	4.16-9.95 x 10^3^/uL
Red blood cell count	4.33	3.96-5.09 x 10^6^/uL
Hemoglobin	13.1	11.6-15.2 g/dL
Hematocrit	41.6	34.9-45.2%
Mean corpuscular volume	96.1	79.3-98.6 fL
Platelet count, auto	282	143-398 x 10^3^/uL
Sodium	141	135-146 mmol/L
Potassium	4.5	3.6-5.3 mmol/L
Chloride	104	96-106 mmol/L
Total CO_2_	23	20-30 mmol/L
Anion gap	14	8-19 mmol/L
Glucose	92	65-99 mg/dL
Creatinine	1.04	0.6-1.3 mg/dL
Estimated GFR	50	GFR >89: Normal, GFR 60-89: Normal to mildly decreased, GFR 45-59: Mildly to moderately decreased, GFR 30-44: Moderately to severely decreased, GFR 15-29: Severely decreased, GFR <15: Kidney failure
Urea nitrogen	22	7-22 mg/dL
Calcium	9.9	8.6-10.4 mg/dL
Albumin	4.2	3.9-5.0 g/dL
Bilirubin, total	0.2	0.1-1.2 mg/dL
Alkaline phosphatase	83	37-113 U/L
Aspartate aminotransferase	30	13-62 U/L
Alanine aminotransferase	13	8-70 U/L
Hgb A1c	5.9	<5.7%
TSH with reflex FT4, FT3	0.94	0.3-4.7 mcIU/mL
Folate	9.7	8.1-30.4 ng/mL
Vitamin B12	157	254-1060 pg/mL
Iron	38	41-179 mcg/dL
Iron binding capacity	326	262-502 mcg/dL
Ferritin	73	8-180 ng/mL
% Saturation	12	%
Urine culture	>100,000 CFU/mL *E. coli*	
	Susceptible to all antibiotics	

The clinical presentation of this patient with clinical evaluation of low MoCA score at 16.5/30 (normal aging score is >26/30), requiring assistance in almost all activities of daily living, and a positive family history of Alzheimer’s disease demonstrate that she has major neurocognitive disorder by Diagnostic and Statistical Manual of Mental Disorders (DSM-V) criteria with functional assessment staging tool (FAST) score 6b [8], indicating moderate-to-severe dementia, likely of Alzheimer’s disease type. Prior to diagnosis, labs and imaging were completed to rule out any other underlying cause. Significant findings were addressed, including vitamin B12 deficiency, which was appropriately repleted with no symptomatic improvement, and the treatment of urinary tract infection, which improved her agitation behaviors. Donepezil was started as first-line treatment for her cognitive decline, and her response was robust only after one week. Her partner informed that he noticed increased mental clarity and the “buzzing” auditory hallucinations were no longer concerns. This raises the question of the role of donepezil along neurological pathways.

## Discussion

One of the leading “top-down” theories for Alzheimer’s disease dementia involves the neurotoxic effects of amyloid beta peptide aggregations. These accumulations cause synaptic disruptions of cholinergic neurons, reducing ACh levels within the brain, specifically within the hippocampus, which lies within the temporoparietal region, the same region that processes auditory sensory inputs [6]. This can explain why Alzheimer's patients have trouble following conversations and eventually impairments in segregating, tracking, and grouping auditory objects over time [6]. Interestingly, even healthy Apolipoprotein E (APOE4) carriers, the strongest marker of genetic susceptibility of developing Alzheimer's disease, demonstrate impairments in interpreting auditory targets [9]. Donepezil aims to increase ACh to make up for the disruptions of cholinergic neurons and increase acetylcholinesterase inhibition to improve cognitive processing efficiency [10].

The “bottom-up” approach starts with sensory input, and in this case, the auditory system is affected. Age-related hearing loss, also known as sensorineural hearing loss or presbycusis, affects one-third of individuals between the ages of 65 and 74, at least 50% of those older than 75 in the United States, and an estimated 430 million people worldwide [11]. Observational studies indicate that the loss of hearing, a sensory quality important in life for communication and survival, leads to reduced quality of life through social isolation and is often associated with cognitive impairments and progression of dementia [6]. While the molecular process is still in exploration, a developing pathway suggests that cholinergic modulation is known to alter auditory frequency responses, promoting synapses at different temporal scales and boosting response gain [6]. ACh is known to be a neuromodulator in the inferior colliculus, the midbrain auditory station where ascending auditory information is processed before traveling to the auditory thalamus and cortex [6].

This patient's years of hearing loss and the development of dementia demonstrates both “top-down” and “bottom-up” neurological pathways impairment. Decades before her dementia diagnosis, she had full workup for her hearing loss with no effective treatment. The current approach to address hearing loss starts with the review of patient's medication list for potential ototoxic agents and empiric trial courses of steroids and antibiotics, but there is no medication for treatment. Most commonly, hearing aids and cochlear implants are prescribed. These devices re-introduce sound, but there are questions if auditory inputs can be properly processed to the brain if the brain itself is not working at its full potential. There is an ongoing randomized, double-blind longitudinal study at Vanderbilt University Medical Center that addresses if donepezil can provide cholinergic enhancement by facilitating cortical reorganization in cochlear implant users, leading to functional improvements in speech recognition and cognition [12].

When hearing is lost, the brain may try to fill in gaps by perceiving sounds that are not truly present, just like the “buzzing” and musical tunes reported in this case. These auditory hallucinations can be as simple as nonspecific sounds in the form of chronic tinnitus, or complex, like musical hallucinations in the form of songs and melodies, also known as auditory Charles Bonnet Syndrome [13]. A study in Brazil is currently undergoing phase 2 trial to determine if those with chronic tinnitus caused by sensorineural hearing loss can improve with donepezil; there are no reports or outcomes disclosed at this time [14]. There are a number of case reports on musical hallucinations. Similarities between the case reports are the observations of risk factors, which include elderly females (>61 years), hearing loss, drugs (primary antihypertensives), brain disease with cerebral atrophy, and social isolation [3,13,15]. Few cases underwent brain imaging that demonstrated atrophy of temporoparietal areas and deactivation of the hippocampus, which point to regions of auditory processing [13]. Interestingly, since musical hallucinations often present in the form of familiar songs from the past, it suggests that the role of memory of the hippocampus is involved [13]. Treatments that were trialed for these case reports often started with anticonvulsants, antidepressants, and antipsychotics that were refractory to treatment. Donepezil and rivastigmine were used with improvement of symptoms; a few reported musical hallucinations vanished completely [13,15].

## Conclusions

The patient in this case report exhibits all the risk factors observed for those with auditory hallucinations. It is unclear if her auditory hallucinations were a result of her hearing loss or a behavioral disturbance of her dementia. Despite the unclear timeline, the effect of donepezil in both “top-down” and “bottom-up” neurological pathways can assist in cognitive function and auditory processing. Donepezil and medications within the same category of acetylcholinesterase inhibitors (galantamine, rivastigmine) should remain as first-line medications due to safer profiles compared to anticonvulsants, antidepressants, and antipsychotics. While the response is notable for this case presentation, further observation and research is needed to demonstrate that donepezil can be more than just a medication for memory.
